# The role of simulation in mixed-methods research: a framework & application to patient safety

**DOI:** 10.1186/s12913-017-2255-7

**Published:** 2017-05-04

**Authors:** Jeanne-Marie Guise, Matthew Hansen, William Lambert, Kerth O’Brien

**Affiliations:** 1 Division of Maternal Fetal Medicine, Department of Obstetrics and Gynecology, Oregon Health and Science University, 3181 SW Sam Jackson Park Road, Portland, OR 97239 USA; 2 Department of Medical Informatics and Clinical Epidemiology, Oregon Health and Science University, 3181 SW Sam Jackson Park Road, Portland, OR 97239 USA; 3 Department of Emergency Medicine, Oregon Health and Science University, 3181 SW Sam Jackson Park Road, Portland, OR 97239 USA; 4 Department of Public Health and Preventive Medicine, Oregon Health and Science University, 3181 SW Sam Jackson Park Road, Portland, OR 97239 USA; 5 Department of Psychology, Portland State University, PO Box 751, Portland, OR 97207 USA

**Keywords:** Health services research, Patient safety, Research design, Patient simulation, Mixed methods, Qualitative research, Pediatrics, Emergency care, National Institutes of Health

## Abstract

**Background:**

Research in patient safety is an important area of health services research and is a national priority. It is challenging to investigate rare occurrences, explore potential causes, and account for the complex, dynamic context of healthcare - yet all are required in patient safety research. Simulation technologies have become widely accepted as education and clinical tools, but have yet to become a standard tool for research.

**Methods:**

We developed a framework for research that integrates accepted patient safety models with mixed-methods research approaches and describe the performance of the framework in a working example of a large National Institutes of Health (NIH)-funded R01 investigation.

**Results:**

This worked example of a framework in action, identifies the strengths and limitations of qualitative and quantitative research approaches commonly used in health services research. Each approach builds essential layers of knowledge. We describe how the use of simulation ties these layers of knowledge together and adds new and unique dimensions of knowledge.

**Conclusions:**

A mixed-methods research approach that includes simulation provides a broad multi-dimensional approach to health services and patient safety research.

## Background

The need to reduce errors and improve patient safety has been recognized as a national priority for over 15 years and has become an important area of investigation in health services research [1–8]. Numerous Academy of Medicine (formerly known as the Institute of Medicine) reports have issued the call for more research and the development of interventions to improve patient safety in emergency services [1–6]. Despite the increased attention and research, as demonstrated by grants and publications, medical errors are still responsible for more deaths than motor vehicle accidents or breast cancer [9]. Rigorous investigations are important to advance knowledge and address these risks, yet are challenging due to the complex and dynamic nature of healthcare.

Patient safety is a continually growing area of health services research. The spectrum of research includes investigations to understand the epidemiology and contributors to adverse events, as well as implementation and testing of interventions to reduce such events. Approaches vary by intended aim and the environment of care. Currently, most critiques and recommendations for strengthening research design in patient safety have focused on implementing interventions to improve education and/or safety [10–12]. In this paper, we describe several research approaches that can be used to investigate the epidemiology and causation of patient safety events.

Two frameworks that commonly underpin patient safety research are Reason’s model for understanding human error [13, 14] and the Donabedian model for evaluating the quality of healthcare [15, 16]. Both are critical to research that seeks to understand causation and contributors to patient safety events. We have previously described a hybrid of Reason’s and Donabedian’s conceptual approaches for patient safety [17]. In this paper, we propose a framework for research that integrates the patient safety framework with mixed-methods research approaches and demonstrate through a worked example how mixed-methods research can be used to build a layered understanding of patient safety.

## Methods

### Applying the consolidated patient safety framework

**Fig. 1:**
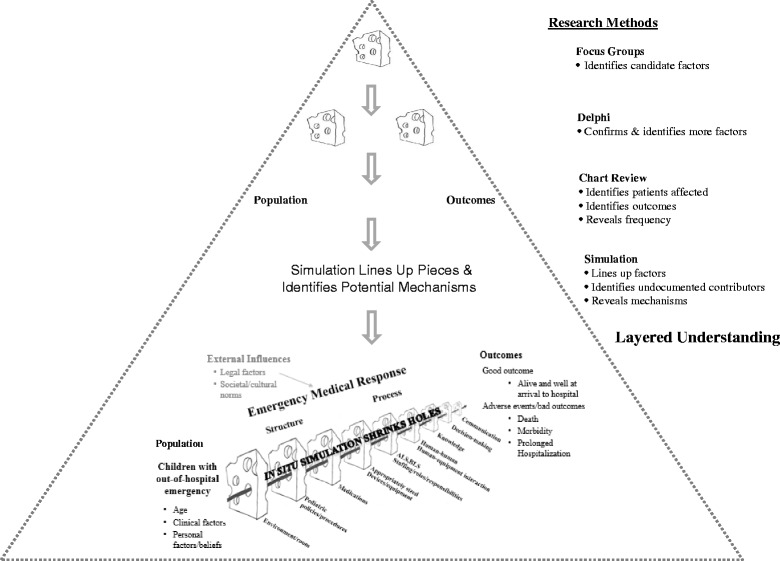
Application of Research Methods to Patient Safety Framework. A consolidated framework for patient safety research is presented which combines Donabedian and Reason’s theoretical models and demonstrates each research approach’s contribution to inform the consolidated patient safety framework (adapted from [17])

We developed a consolidated framework for patient safety research that integrates Reason’s and Donabedian’s patient safety models with mixed-methods research approaches (Fig. 1). Gathering the data to empirically demonstrate how well a theoretical framework performs is essential. The investigative team assessed the performance of the consolidated framework for patient safety research through application in the Children’s Safety Initiative. The Children’s Safety Initiative-Emergency Medical Services (CSI-EMS) is a large multi-year NIH-funded study (1R01HD062478) aimed at understanding the epidemiology and predictors of patient safety in the prehospital emergency care of children. In-hospital and out-of-hospital health care are complex adaptive systems consisting of multiple parts whose dynamic nature and complexity make research challenging. Pediatric patients pose additional challenges that may further increase the risk of medical errors. Factors such as a wide spectrum of medication dosing due to the size variation of children, the inability of young children to provide a medical history or clearly communicate complaints, and physical and developmental characteristics all may affect treatment strategies and pose challenges to providers [18].

While the incidence of, and contributors to, adverse events in hospitals are well described, [19, 20] the nature of adverse events and associated circumstances in the prehospital care environment are largely unknown, particularly in relation to children [4]. Given the complexity of issues relating to prehospital pediatric emergency care, a thoughtful, broadly-based and scientifically rigorous approach is required to inform patient safety in this context. The CSI-EMS applied a mixed-methods approach with four main study components: focus groups, Delphi process, chart reviews, and *in situ* simulations. As shown in the Fig. 1, each research component works together to build a broad and rich understanding of individual and systems contributors to the occurrence of patient safety events. The study was approved by Oregon Health & Science University’s Institutional Review Board (IRB # 00006942). We briefly describe the strengths and limitations of each selected component, highlight the important and complementary role that simulation plays in informing gaps, and demonstrate the rich understanding each dimension brings to our understanding through the consolidated patient safety framework.

## Results

The investigative team successfully carried out the various research approaches of the CSI-EMS study and assessed how each phase of the study integrated within the consolidated patient safety framework. Details of strengths and limitations of each study design relative to applied patient safety research and the role of simulation are discussed.

### Focus groups

Because little was known about out-of-hospital pediatric patient safety, our research started with focus groups. Focus groups are often adapted from grounded theory approaches and are therefore well suited to the exploration of new subject areas. In mixed methods research, focus groups are often used for hypothesis generation. Investigators assemble groups of individuals whose experiences and perspectives inform the topics of interest. The groups discuss these topics with the guidance of a moderator and themes are brought together through qualitative thematic analysis to begin to understand a topic [21, 22]. In our study, after signing written consent, focus groups were held among paramedics, emergency department providers, and staff from differing practice environments to gain an initial working understanding of the important factors in pediatric patient safety in out-of-hospital care.

#### Strengths

The strengths we found in the focus group approach include: that it was useful to develop an initial set of factors and processes perceived to be associated with safety events and suboptimal outcomes; it promoted a collective appraisal by participants in the relative importance of contributors to safety events; and it created a shared terminology or dictionary of terms that would be relevant and meaningful to the practitioners in the area [23]. An important example of the value of focus groups as a starting point arose around terminology and differing mental models. In the hospital setting, the term “safety events” is commonly understood to refer to patient safety events; however, through focus group discussions we found that the prehospital environment, particularly emergency medical services more commonly understand “safety events” as referring to the physical safety of providers, occupational safety, and motor vehicle safety of ambulances rather than patient safety. Furthermore, because little was known about patient safety in the prehospital environment, we found that a modified grounded theory approach which allowed discoveries to emerge from the focus groups rather than trying to prove a specific hypothesis true or false provided the optimal starting point [24, 25].

#### Limitations

There are limitations to focus groups which include: lack of representativeness due to small sample sizes and potentially limited geographic area; potential for dominance by some participants or suppression of the expression of opinions by others (which can be managed by trained facilitators); saturation (redundancy of emerging theme) may not be achieved if too few focus groups are conducted; and the need to use skilled moderators and qualitative analysts to minimize bias. Despite these limitations focus groups can provide an excellent starting point for mixed-methods investigations. Group dialogue and the opportunity it provides for elaboration is particularly useful when seeking to understand the multiple dimensions of a complex problem such as the delivery of prehospital pediatric care.

#### Integration with simulation

While focus groups are helpful to form an initial understanding of team interactions and the function of systems of care that are not easily measured quantitatively, simulation offers a way to take the hypotheses generated from these formative research approaches and quantitatively test specific elements of in a controlled environment with validated measures.

### Delphi process

The Delphi process [26–28] is a particularly robust research method to apply when consensus opinion is important. It involves the sequential administration of surveys (traditionally 3) to a defined group of individuals where each survey round informs the next and the ultimate product is consensus. Our initial focus group work provided the formative foundation for the content of the initial Delphi survey. While Delphi processes are often conducted within small groups in order to maintain participation through several survey rounds, our Delphi was conducted on a national scale and is one of the largest Delphi processes conducted in any field. In order to support a national survey process, we conducted the Delphi through a computerized survey (also referred to as “e-Delphi method”). Our objective was to achieve global consensus among EMS providers from various regions of the United States on the most important contributors to patient safety events in the out-of-hospital care of children’s emergencies [29]. After the initial round, the questionnaire items in the two subsequent survey rounds were informed by results of each prior cycle, as per traditional Delphi methods.

#### Strengths

In our experience, the Delphi process contributed many insights and demonstrated multiple strengths: the online surveys provided an anonymous forum for input from individuals who are independent of one another (removing concern for the dominance of an individual); the iterative process developed consensus on the relative importance of various factors (which is often a key question for patient safety and research in general); the format of the e-Delphi process allowed large numbers of people to be included (our CSI-EMS process was among the largest Delphi processes conducted to date with 753 participants from across the United States); and, the increasingly more frequent use of an electronic web-based platform for Delphi studies promotes recruitment and retention of a national or even international sample.

#### Limitations

We found that the Delphi process identified several unexpected and rare factors that likely would not have been captured in smaller focus groups or in-person settings (for example discussions about emotional factors impeding safety and decision-making and issues with the patient safety culture). However, potential limitations of the method include: self-selection for participation (in our case this was counteracted by national recruitment and large number of participants); potential for high attrition across later rounds (Delphi requires that the same group participate across all rounds); and the analysis and feedback into sequential rounds of surveys requires sophisticated skills in both quantitative and qualitative data and efficient turn-around. Even so, the Delphi method has many strengths and its capacity for the electronic format is promoting the larger sample sizes that are often sought for rigorous research.

#### Integration with simulation

The Delphi process generated a ranked list of perceived major contributors to patient safety allowing us to prioritize topics for investigation. These topics were included in our simulation scenarios. For example, in the CSI-EMS the Delphi process identified airway management and provider anxiety as two of the most important contributors to patient safety. We designed simulations to further explore how these factors play out in clinical care.

### Chart reviews

Chart review processes are familiar to many clinical investigators. In the case of the CSI-EMS, we developed and revised an existing highly-respected chart review tool used in the hospital setting (used in the Harvard Medical Study) [8, 19]. We trained a panel of paramedics and emergency medicine physicians using a standardized protocol to review charts and identify safety events during prehospital care given by paramedics and emergency medical technicians responding to pediatric emergency calls.

#### Strengths

Strengths of chart reviews include: a reflection of actual clinical practice in a real-world setting; a systematic approach to uncover information through the use of structured chart review tools; and a process that supports uniformity and objective reviews, standardization between observers, and verification by others, all of which reduce the potential for bias.

#### Limitations

Although chart reviews can be very helpful as a true reflection of clinical practice, they have many limitations. Reviewing charts requires considerable time and expense; charts contain restricted confidential information which poses a potential, but minimal, risk to patient confidentiality; charts are completed with the intent to document and manage care rather than to provide research data, and therefore there can be issues of missing, insufficient and conflicting information; and there can be inter- and intra-observer variability among expert reviewers requiring the establishment of rigorous processes and standardized tools. In summary, the major limitations of chart review involve its time-consuming nature, both in the design and conduct needed to ensure a rigorous and objective processes, and the expense required to compensate for the time-consuming task. However, the fact the charts are a direct reflection of real-world clinical care is a major strength for understanding and improving patient safety in clinical care.

#### Integration with simulation

Results from a chart review process can be tightly integrated with simulation. Charts can identify representative clinical cases whose elements can be integrated into simulation scenarios. In the case of the CSI-EMS, using the input of the Delphi that identified airway management as a potentially important contributor to safety events, and chart reviews that identified the age of a child as a major feature of clinical management, we selected cases where there were airway management errors to develop simulation scenarios based on actual charts. Additionally, the chart reviews may generate causal hypotheses which can then be tested in the simulated setting (e.g., management of similar scenarios differs for infant vs. school-age children).

### Simulation

Simulation is most commonly used for educational purposes and team training. The potential for simulation as a research tool, however, extends beyond the educational paradigm. We used simulation to understand how the pieces of errors and issues fit together, in what sequence, and to understand the undocumented underlying processes and contributors.

#### Simulation design

Simulation scenarios were developed based on high-risk clinical cases and contributors identified from focus group, Delphi, and chart review stages of the study. As an example, trauma and cardiac arrest were found to be high-risk clinical conditions and younger age was a significant contributor. Pediatric trauma and cardiac arrest simulation scenarios were developed by clinicians, programmed into the simulator, pilot tested, and revised following pilot testing. Moulage was used to increase the realism of the manikins (simulating bruising, bleeding, etc.), props to decorate the scenes (e.g. vehicles and strollers for accidental trauma; used pizza boxes, fast food wrappers, alcohol bottles for disheveled appearance of home), and hired professional actors to play the role of confederates (tearful concerned mother for accidental trauma, suspicious boyfriend for non-accidental, etc.) to increase the realism of the scenarios and closely resemble clinical care. Participants were EMS teams who responded to the simulated cases *in situ* in the same manner they do in actual practice, dispatched through central dispatch and driving to the scene in fire trucks and ambulances with lights flashing. All scenarios were video-recorded for subsequent blinded review. Reviewers used the validated CTS™ tool as well as a newly created technical performance assessment tool developed by clinical experts based on expert consensus of best practice and existing EMS protocols for the specific scenarios.

#### Strengths

Table 1 summarizes strengths and limitations of simulation as a research tool. Simulation provides a unique opportunity to highly control the environment, scenario, and other features that are often unpredictable and inconsistent in real-life clinical care. It is as close as one can come to creating a laboratory setting for clinical care. This allows investigation of mechanisms with the ability to manipulate specific factors of interest. Investigators can conduct systematically vary 1 or 2 factors at a time and obtain detailed measurements of the process and results. Furthermore, the ability to capture all scenarios by video provides a format for independent raters who are blinded to the study hypotheses, individuals, and even timing of interventions (in the case of before-after studies) to record data and observations – reducing bias. In addition, simulation can provide objective measurements during the provision of care, for example through physiologic recordings and/or serum or other samplings.

**Table 1:** Summary of the Strengths and Limitations of Simulation as a Research Tool

Strengths
Can be linked to other patient safety research methods to deepen understanding
Can be used to recreate events that are difficult to observe due to rarity or complexity
The environment is controlled and cases are standardized and reproducible
Hypotheses related to patient safety events can be tested while reducing confounding
Outcomes can be measured using validated tools
Challenges and Limitations
The spectrum of events that can be studied is limited to the fidelity of the mannequins
Technical problems with simulation equipment are possible and can invalidate a study scenario
Technical expertise with the equipment and common simulation problems is needed
Practical Tips for Success
The fidelity of the equipment and environment need to be carefully considered in study design
Scenario fidelity can be greatly enhanced by using trained confederates
Cases should be prepared with the assistance of an experienced simulation technician
Cases should be pilot tested to identify weaknesses in the scenario and equipment

Simulation can be a highly effective tool for patient safety research as it allows investigators to re-create rare events or specific complex scenarios in a controlled environment. This can be particularly useful for understanding the mechanism by which observed findings occur and for testing hypotheses of association and causation. For the CSI-EMS, simulation allowed us to re-create the rare scenarios of pediatric prehospital cardiac arrest and severe pediatric trauma and objectively test whether key factors or variables changed the likelihood of errors occurring during care. In addition, it allowed us to study procedural proficiency and decision-making relating to pediatric out-of-hospital airway management.

#### Limitations

Simulation has important limitations that need to be considered before use. It is a costly research tools not only because of the expense of simulators but also because of the human capital required. Simulators used to replicate clinical care often have highly sophisticated computers requiring unique skills to run. They are still largely in their infancy and because of this have limitations in fidelity to the human condition. As an example, it is difficult to replicate limpness, flexibility, cyanosis, or temperature, which are important considerations in clinical diagnoses. Because clinical care is most often delivered in clinical teams, research teams using simulation may need to play supporting roles which may require clinical knowledge and requires the team to consider the degree that scripting of these roles affects results or introduces bias.

## Discussion

Simulation provides the greatest benefit to patient safety research when it is part of a larger conceptual framework. Through this application to research we demonstrate that the hybrid conceptual framework is a useful model for investigators using simulation for patient safety research and allows logical integration with other elements of a mixed-methods approach. As discussed in this paper and shown in the Fig. 1, focus groups generate elements that contribute to patient safety (e.g., upset family members, bystanders, external conditions); the Delphi process prioritizes the most important factors and enriches the understanding of factors (e.g. stress from seeing young child hurt, upset family members); the chart review ties these factors together with patient outcomes and clinical features (e.g. child trauma, cardiac arrest in young child); and simulation allows an exploration of the structure and processes that allow the holes in the “Swiss cheese” analogy to line up and cause poor outcomes.

As a research tool, simulation is limited by the resources (human, technological, and monetary) required to conduct scenarios, and usually is limited in the numbers of potential participants. Given this, investigators should carefully design scenarios and consider tests of specific hypotheses, informed by statistical power considerations that address the most important safety issues. Robust scenario selection, development of objective data collection instruments, and careful consideration of the analytic approach are essential to conducting high-quality simulation research. Even with video capture, careful consideration for variables that will be measured, testing of the instruments ability to capture these, ensuring objective independent assessments when possible, and assessing inter-rater reliability among evaluators are important a priori considerations.

## Conclusions

Patient safety is a dynamic and complex area of health services research with multiple components and factors to consider [30]. Investigating dynamic multi-component complex events can be a daunting challenge, especially in the highly variable context of prehospital care. We provide a consolidated patient safety framework that integrates simulation into mixed-methods research. This framework integrates what is known in human factors and patient safety with what is known about the strengths and limitations of research designs. We have demonstrated the viability of this approach through a large multi-year investigation in a new area of patient safety research, describe our reflections on the strengths and limitations of several common research approaches, and highlight the ways that these different approaches address limitations in each other. While greater testing is needed, simulation holds promise as a tool to bridge gaps from other research approaches. Simulation provides a laboratory-type environment that facilitates the testing of hypotheses, and can confirm findings arising from other study types, thus promoting a comprehensive understanding of research topics.

## Abbreviations

AHRQ: Agency for Healthcare Research & Quality
CSI-EMS: Children’s Safety Initiative-Emergency Medical Services
IOM: Institute of Medicine
NIH: National Institutes of Health

## References

[1] Institute of Medicine (1993). Emergency medical services for children.

[2] Institute of Medicine (2001). Crossing the quality chasm: a new health system for the 21st century.

[3] Institute of Medicine (2003). Patient safety: achieving a new standard of care.

[4] Institute of Medicine (2006). Emergency medical services at the crossroads.

[5] Institute of Medicine (2007). Emergency care for children: growing pains.

[6] Kohn L, Corrigan J, Donalson M (1999). To err is human: building a safer health system.

[7] Leape LL (1994). Error in medicine. JAMA.

[8] Leape LL, Brennan TA, Laird N (1991). The nature of adverse events in hospitalized patients: Results of the Harvard Medical Practice Study II. N Engl J Med.

[9] Leistikow IP, Kalkman CJ, Bruijn H (2011). Why patient safety is such a tough nut to crack. BMJ.

[10] Campbell M, Fitzpatrick R, Haines A (2000). Framework for the design and evaluation of complex interventions to improve health. BMJ.

[11] Haji FA (2014). From bricks to buildings: adapting the medical research council framework to develop programs of research in simulation education and training for the health professions. Simul Healthc.

[12] Shekelle PG, Pronovost PJ, Wachter RM (2011). Advancing the science of patient safety. Ann Intern Med.

[13] Reason J (1990). Human error.

[14] Reason J (2000). Human error: models and management. BMJ.

[15] Donabedian A (1988). The quality of care: how can it be assessed?. JAMA.

[16] Donabedian A (2005). Evaluating the quality of medical care. Milbank Q.

[17] Guise JM, Mladenovic J (2013). In situ simulation: identification of system issues. Semin Perinatol.

[18] Woods D, Thomas E, Holl J (2005). Adverse events and preventable adverse events in children. Pediatrics.

[19] Brennan TA, Leape LL, Laird NM (1991). Incidence of adverse events and negligence in hospitalized patients. N Engl J Med.

[20] Thomas E, Studdert PM, Burstein HR (2000). Incidence and types of adverse events and negligent care in Utah and Colorado. Med Care.

[21] Morgan DL (1988). Focus groups as qualitative research.

[22] Morgan DL (2013). Integrating qualitative and quantitative methods: a pragmatic approach.

[23] O’Brien K (1993). Using focus groups to develop health surveys: an example from research on social relationships and AIDS-preventive behavior. Health Educ Q.

[24] Corbin J, Strauss A (2008). Basics of qualitative research: techniques and procedures for developing of grounded theory.

[25] Creswell JW, Plano-Clark VL (2007). Designing and conducting mixed methods research.

[26] Hasson F, Keeney S, McKenna H (2000). Research guidelines for the Delphi survey technique. J Adv Nurs.

[27] Jones J, Hunter D (1995). Consensus methods for medical and health services research. BMJ.

[28] Dalkey NC. The Delphi method: an experimental study of group opinion. Rand. 1969;ii-79. http://www.rand.org/content/dam/rand/pubs/research_memoranda/2005/RM5888.pdf.

[29] Guise JM, Meckler G, O’Brien K (2015). Patient safety perceptions in pediatric out-of-hospital emergency care: Children’s safety initiative. J Pediatr.

[30] Weick KE, Sutcliffe KM (2001). Managing the unexpected assuring high performance in an age of complexity.

